# Risk of venous thromboembolism in patients with COVID-19 during 2020; a retrospective cross-sectional study in a Swedish health care system

**DOI:** 10.1038/s41598-023-32637-x

**Published:** 2023-04-04

**Authors:** Jens Wretborn, Matthias Jörg, Patrik Benjaminsson Nyberg, Daniel B. Wilhelms

**Affiliations:** Department of Emergency Medicine and Department of Biomedical and Clinical Sciences, Linköping University, 581 85 Linköping, Sweden

**Keywords:** Thromboembolism, Viral infection, Risk factors

## Abstract

To establish the impact of COVID-19 on the pre-test probability for VTE in patients with suspected VTE. This was a retrospective, observational, cross-sectional study of patients 18 years and older undergoing diagnostic tests for VTE in an integrated healthcare system covering a population of 465,000 during the calendar year of 2020. We adjusted for risk factors such as age, sex, previous VTE, ongoing anticoagulant treatment, malignancy, Charlson score, ward care, ICU care and wave of COVID-19. In total, 303 of 5041 patients had a positive diagnosis of COVID-19 around the time of investigation. The prevalence of VTE in COVID-positive patients was 10.2% (36/354), 14.7% (473/3219) in COVID-19 negative patients, and 15.6% (399/2589) in patients without a COVID-19 test. A COVID-positive status was not associated with an increased risk for VTE (crude odds ratio 0.64, 95% CI 0.45–0.91, adjusted odds ratio 0.46, 95%CI 0.19–1.16). We found no increased VTE risk in COVID-positive patients. This indicates that COVID-19 status should not influence VTE workup.

The study was pre-registered on May 26, 2020 at ClinicalTrials.gov with identifier NCT04400877.

## Introduction

### Background

In the ongoing COVID-19 pandemic reports have shown an increased risk of VTE, including both pulmonary embolism (PE) and deep venous thrombosis (DVT)^1–5^ and international guidelines recommend prophylactic anticoagulation for all hospitalized patients with COVID-19^6^. The majority of the initial reports on VTE in COVID-19 have been carried out in the intensive care unit (ICU) and show a prevalence of VTE of 20–30%^1–3^. This is higher compared to cohorts of non-selected ICU patients, in whom the prevalence of VTE is closer to 10%^7–10^. However, studies on ICU patients with severe sepsis and viral infections like H1N1 influenza have shown a prevalence of VTE of 37% and 44%, respectively^11,12^. The prevalence of VTE in hospitalized non-ICU patients with COVID-19 is 3–4%^13–15^, similar to studies on internal medicine patients with prophylactic anticoagulation^16^. However the VTE risk and prevalence in outpatients with known or suspected COVID-19, is less studied^17^.

In a recent large nationwide cohort study from Sweden, Katsoularis et al.^18^ found a relative risk increase for VTE in patients with COVID-19. However the study only adjusted for chronic medical conditions and not for other concomitant acute illnesses. Since the inclusion was any type of contact with the Swedish health care system it is likely that the COVID-positive was a select group with an acute upper respiratory illness which is a known risk factor for VTE^16,19^.

In isolated outpatient cohorts, no increase in the risk of VTE has been found for patients with COVID-19. Freund et al., studied PE prevalence in patients undergoing computed tomography pulmonary angiography (CTPA) in 6 Emergency Departments (ED) in Europe and found no increased prevalence, or risk, of VTE in patients with COVID-19. Similarly, Thoppil et al.^20^ found no increased risk of VTE for COVID-positive patients in a retrospective observational trial of 27,051 patients in the United States. We have previously investigated the prevalence and risk of VTE in patients with and without COVID-19 in a regional healthcare system in Sweden during the first wave of the pandemic (March through May 2020). In that period, we found no significant increase in prevalence or risk for VTE but the number of COVID-positive patients was low and a larger cohort is needed to validate the results^21^.

Due to the alarming reports of high prevalence of VTE during the beginning of the pandemic, most patients with COVID-19 will be considered for potential VTE as outpatients in the ED, regardless whether they are admitted to hospital or discharged home. Hence an informed risk assessment is imperative to perform a reasonable workup while limiting the risk of overuse of health care resources on a system level.

### Goals of this investigation

To investigate if COVID-19 was associated with an increased risk of VTE in patients undergoing testing for VTE in a regional healthcare system in Sweden during the calendar year of 2020.

## Methods

### Study design and setting

In this retrospective observational study we have evaluated the risk and prevalence of VTE during the calendar year of 2020 in the county of Östergötland (Region Östergötland, Sweden). The county has a population of 465,000 (December 31, 2019) and healthcare is provided by a central, publicly funded healthcare system. There is one rural community hospital, one urban community hospital and one academic tertiary care hospital. All hospitals, outpatient clinics and primary care centers in the county use the same electronic health records (EHRs) and all diagnostic studies of VTE are performed within the healthcare system.

### Selection of participants

Adult patients (≥ 18 years of age) who underwent a diagnostic test for suspected VTE during the calendar year 2020 were included. Follow up investigations, tests performed on referred patients from another healthcare system, and planned but non-performed tests were excluded.

### Exposures

The exposure was COVID-19 infection, defined as a positive PCR test up to 14 days prior to or 7 days after the diagnostic test for VTE. This timeframe was chosen a priori to account for the delay from symptom onset to deterioration^22^ and delay to PCR test in the beginning of the pandemic. All patients with at least one diagnostic test for VTE during 2020 were matched with the regional SARS-CoV-2 database of real time PCR results. PCR was the diagnostic criteria for COVID-19 used in our system and patients with high probability for COVID-19 despite a negative PCR were tested repeatedly. PCR data was extracted from the healthcare system’s central diagnostic laboratory, the only authorized SARS-CoV-2 laboratory during this period.

### Measurements

Additional known risk factors for VTE were extracted from the EHR. Subgroup analysis of outpatients and patients in the ward or ICU based on date of investigation was performed to distinguish prevalence of VTE from COVID-19 infection, and from severe disease^7,11^. In-hospital care on a ward or in the ICU was defined as a minimum of 24 h of care, as tests related to the ED presentation may be deferred up to 24 h. Anticoagulant treatment was defined as treatment with any B01A class drug^23^ more than seven days prior to the diagnostic test. Risk factors for VTE were; age (continuous), sex (male/female), previous VTE (yes/no), malignancy (yes/no), ward care (yes/no), intensive care (yes/no) and Charlson score (continuous) were extracted from the EHR. The Charlson score was calculated from previous diagnosis registered in the EHR, which has partial coverage of diagnoses prior to 2008 and full coverage thereafter. Mortality at 30 days was defined as all-cause mortality based on the Swedish national civil registration registry.

### Outcomes

The outcome was a diagnosis of VTE by CTPA or ultrasound. Written study reports were extracted from the picture archiving and communication system for all CTPA and ultrasound for deep venous thrombosis (DVT). Findings of PE were coded as positive for any contrast defect in a subsegmental, or more central pulmonary artery. Any additional finding classified as definitive or probable PE by the attending radiologist was coded as PE positive. DVT was diagnosed with complete compression ultrasound or 3-point compression ultrasound of the leg^24^. Isolated muscle vein thrombosis and thrombophlebitis were classified as negative examinations. Patients with multiple tests of the same modality, on the same day were classified as duplicates and combined to a single test. The reports were classified as positive or negative independently by JW, JA and MJ. Ambiguous reports and differences in classification were reviewed by JW, MJ and PBN and solved through full consensus. A subset of diagnostic tests were done in the department of clinical physiology and were already classified as positive or negative.

### Analysis

Descriptive data was reported as percentage, mean with standard deviation (SD) or median with interquartile ranges (IQR). Prevalence was reported by separate diagnoses, e.g. the prevalence of PE was CTPA positive tests compared to all performed CTPA. Prevalence was compared with the chi-squared test with pre-defined subgroup analysis for PE and non-PE VTE, mainly DVT or Fisher’s exact test for small groups. Patients with no RT-PCR test results in 2020 were treated as a separate subgroup to avoid bias based on test availability. Differences in distributions of PE were assessed with the Kruskal–Wallis test. Logistic regression was used to analyze the crude and adjusted odd ratios (OR) for a VTE by COVID-19 status and to account for the different waves of COVID-19 during 2020.

A sample size calculation with the Chi-square goodness of fit test for two categorical variables with a conservative effect size of 0.1 (alpha 0.05, power 0.8) required 785 samples. Based on an expected test rate of 500 diagnostic studies per month, three months of data was included in our previous study^21^. No additional power calculation was made for this study. Data was imported into Pandas (v 0.23)^25^ and analyzed with Python using the Scipy library (v 1.17)^26^ and Statsmodels library (v. 0.12)^27^.

### Ethical considerations

This study was carried out in accordance with The Declaration of Helsinki^28^. Ethical approval was granted by the Swedish Ethical Review Authority with permit reference 2020-02701. Informed consent was waived by the review authority. The study has been conducted according to the STROBE guidelines for reporting observational trials.

## Results

During the calendar year of 2020, 5401 patients were investigated for possible VTE on 6169 occasions in the Region Östergötland health care system of which 303 had a positive diagnosis of COVID-19 based on laboratory PCR data on 354 occasions. The COVID-positive patients were more often male, admitted to hospital or ICU and were more likely to receive invasive ventilation or die (Table 1). The prevalence of previous malignancy and treatment with anticoagulants were lower in the COVID-positive group while a diagnosis of previous VTE was higher. There were more investigations done for PE in 2020 compared to the five previous years (3425 vs mean 2662).

**Table 1:** Demographic data of included patients.

	COVID-positive	COVID-negative or untested
n	303	5098
Age	62.9	64.4
Male sex	159 (52.5%)	2186 (42.9%)
BMI	29.2 (n = 132)	28.6 (n = 2389)
Anticoagulant treatment	41 (13.5%)	1698 (33.3%)
Previous VTE	32 (10.6%)	349 (6.8%)
Previous malignancy	46 (15.2%)	973 (19.1%)
Charlston score (IQR)	3.00 (1.00—5.00)	3.00 (2.00—5.00)
Positive VTE	32 (10.6%)	774 (15.2%)
Positive PE	29 (9.6%)	415 (8.1%)
Admitted to a ward	126 (41.6%)	1310 (25.7%)
Ongoing ward care	97 (32.0%)	748 (14.7%)
Admitted to an ICU	19 (6.3%)	98 (1.9%)
Ongoing ICU care	19 (6.3%)	18 (0.4%)
Ventilator treatment	23 (7.6%)	55 (1.1%)
30 day mortality	9 (3.0%)	35 (0.7%)

The prevalence of PE was lower in COVID-positive patients compared to negative or untested patients (p = 0.02), and lower but not statistically significant (p = 0.32) in patients with DVT (Table 2). There was no difference in distributions of thrombosis within the pulmonary arteries between negative and untested patients, and COVID-positive patients (p = 0.1) (Table 2).

**Table 2:** Prevalence of positive examinations by type of venous thromboembolism and COVID-19 status.

	COVID-positive (%)	COVID-negative	COVID unknown
Venous thromboembolism	10.2% (36/354)	14.7% (473/3224)	15.4% (400/2591)
Deep venous thrombosis	8.6% (5/58)	13.1% (168/1281)	14.4% (246/1713)
Pulmonary embolism	10.7% (32/300)	15.6% (308/1974)	17.5% (159/908)
Central	6.3% (2/32)	15.6% (48/308)	13.8% (22/159)
Lobar	40.6% (13/32)	35.1% (108/308)	40.9% (65/159)
Segmental	40.6% (13/32)	37.3% (115/308)	31.4% (50/159)
Subsegmental	12.5% (4/32)	12% (37/308)	13.8% (22/159)

The prevalence of VTE was higher in patients with COVID-19 treated in the ICU compared to COVID-negative or untested patients but not statistically significant (p < 0.14). In contrast, it was lower in both the ward and outpatient cohort although the association was only statistically significant for the outpatient group (p = 0.02). Further dividing the outpatient group to patients being admitted to the ICU or ward within 24 h from outpatient VTE testing, the prevalence of VTE was lower in the COVID-19 group compared to the negative and untested (Table 3).

**Table 3:** Prevalence of venous thromboembolism by disposition and COVID-19 status.

	COVID-positive	COVID-negative	COVID unknown	p
ICU	31% (8/26)	6% (1/17)	6% (1/18)	0.14*
Ward	10% (10/104)	15% (84/573)	14.% (47/342)	0.48**
Outpatient	8% (18/224)	15% (388/2634)	16% (352/2231)	0.02**
ICU Admission	4% (1/24)	14.% (13/92)	4% (1/23)	0.30*
*Ward Admission	9% (12/137)	21% (231/1101)	31% (123/397)	< 0.001 **

*Fisher exact (COVID-positive vs COVID-negative).

** Chi-square.

The unadjusted OR for a positive diagnostic test of VTE when positive for COVID-19 was 0.64 (95%CI 0.45–0.91). When adjusting for potential confounders the OR was further decreased to 0.46 (95%CI 0.19–1.16). Previous VTE was the only factor which significantly increased the risk for VTE (OR 4.58, 95%CI 2.76–7.62) while age (OR 1.01, 95%CI 0.99–1.03) and ICU (OR 1.10, 95%CI 0.39–3.09) had a non-significant association. Ongoing anticoagulation treatment significantly reduced the risk for VTE with an OR of 0.55 (95%CI 0.43–0.71).

**Figure 1:**
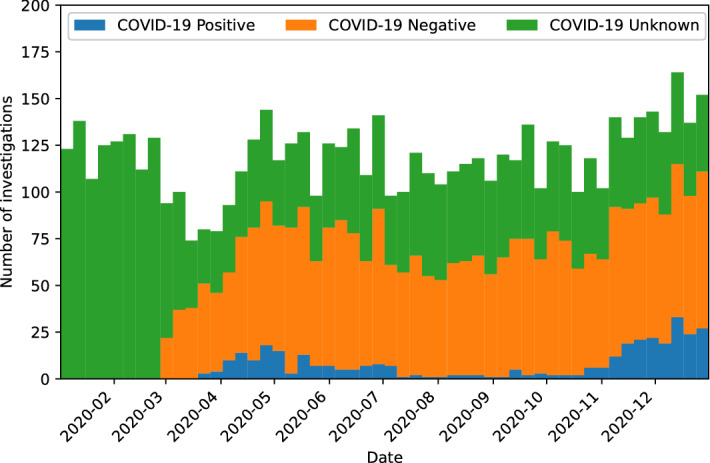
Incidence of diagnostic tests for venous thromboembolism by COVID-19 status.

When adjusting for COVID-19 wave as adjudicated by the Swedish Ministry for Health and Welfare, the first wave (2020-03-01 to 2020-09-30) and second wave (2020-10-01 to 2020-12-31) of the pandemic had non-significant increases of risk for VTE with ORs of 1.02 (95%CI, 0.5–2.1) and 1.09 (95%CI, 0.5–2.4) respectively. The number of investigations for VTE had a nadir in the beginning of the pandemic and increased to similar pre-pandemic levels during the first wave and was higher during the second wave (Fig. 1).

## Limitations

This was a retrospective observational study and the results are limited to the variables we were able to control for. We used commonly accepted risk factors for PE and DVT defined by Wells et al.^29,30^ but were not able to obtain physiologic data or referring physician’s assessment at the time of the diagnostic test to adjust for all criteria in the conventional diagnostic tools^30,31^. However, we believe that we have been able to adjust for the majority of confounders for VTE when evaluating infection with SARS-CoV-2 as a possible risk.

Only patients who had a diagnostic test for possible VTE were included in this study while we did not include patients ruled out by other means, like d-dimer, hence we cannot calculate the prevalence for all patients considered for VTE. However there was no increased prevalence of VTE in the untested cohort except for patients in intensive care. If there was a true increase of VTE from COVID-19, irrespective of other risk factors, we would expect to see an increase in VTE as the incidence of COVID-19 was rising in the community.

SARS-CoV-2 status was missing from a large proportion of patients undergoing testing for VTE in 2020 which limits the generalisability of the results. We only had the ability to adjudicate the patients’ COVID-19 status based on PCR testing, which was the accepted method in our healthcare system. The incidence of investigations with COVID-19 status unknown was fairly constant during the pandemic (Fig. 1) which likely limits systematic bias of the results.

We may have missed a few PEs by not including chest CT or scintigraphy. However, most findings associated with PE on chest CT would likely warrant a definitive workup including CTPA in our health care system as CT chest is not considered diagnostic for VTE. Scintigraphy is rarely performed in our system and we believe that this would not influence the results. We based the classification of PE on the radiology report and not an independent read of the image data. While this may have introduced a subjective interpretation, it reflects the actual practice in Sweden where treating physicians rely on the radiology report for the diagnosis of PE.

## Discussion

An infection with SARS-CoV-2 virus confirmed on RT-PCR was not associated with an increased risk, or prevalence of VTE in patients undergoing a diagnostic test for VTE in a large integrated healthcare system in Sweden. This study confirms our findings from the first three months of the SARS-CoV-2 pandemic^21^ and concurs with the results from a large retrospective observational study from the US by Thoppil et al.^20^ which compared the prevalence and risk of VTE in ambulatory ED patients with and without COVID-19. Additionally Freund et al.^32^ found no association between confirmed diagnosis of COVID-19 and a PE in 3253 patients undergoing CTPA for suspected PE in the ED. However there was a high prevalence of VTE in patients with confirmed COVID-19 in the ICU in our study, similar to previous reports^1–3^, and similar to previously established levels of VTE in ICU patients with severe infections caused by other pathogens^11,12^.

Studies have attributed the reportedly high VTE rates in COVID-19 to conventional VTE, as well as in-situ immunothrombosis^33^. If the VTE rate in COVID-19 were driven by immunothrombosis in other than the most severe cases, however, we would expect an increased proportion of patients with peripheral clots in segmental or subsegmental pulmonary arteries^34^. This was not the case in our study, where the distribution of pulmonary embolisms was similar in both groups (Table 3). This further underlines the fact that, rather than focusing on COVID-19 as an independent risk factor for VTE, we should instead consider disease severity which has a well-established, positive correlation to pre-test probability of a VTE.

In a large nationwide study in Sweden, Katsoularis et al.^18^ found an increased relative and absolute risk of VTE in a cohort of patients with SARS-CoV-2 positive PCR tests compared to an undifferentiated cohort of patients with any type of contact with the Swedish healthcare system. It is reasonable that an upper respiratory virus with the potential to cause severe pneumonia will cause a small increase in baseline risk compared with any, or potentially no acute illness, as both acute illness and severity of acute illness is associated with increased prevalence of VTE^7–9,11^. However, this does not necessarily need to increase the predictive value of an airway infection per se in a more select population of patients, like patients deemed at risk for VTE. Hence the results by Katsoularis et al., do not contradict the results of this study or previous studies performed in the ED^20,32^.

There was an increase in the number of investigations of pulmonary embolism compared to the previous years in our healthcare system (3425 vs mean 2662). Together with the decreased OR for COVID-19 this may indicate overtesting for pulmonary embolism in patients with COVID-19. Computer tomography has been suggested as a modality to risk stratify patients with COVID-19 and providers may have deemed a CTPA convenient to risk-stratify and workup the patients for potential PE. With scarce but concerning data regarding VTE in COVID-19 during the beginning of the pandemic it was arguably reasonable to have a high degree of suspicion and a low threshold for VTE workup, which is likely reflected in high numbers of investigation. However, with accumulating data failing to show an increased risk of VTE in patients with COVID-19 investigated for VTE^20,21,32^, we question whether the increase in radiation and the added risk of contrast use is justified onwards.

In summary, testing positive for COVID-19 did not increase the OR for VTE compared to a negative test when adjusting for known risk factors and ongoing treatment with anticoagulation. This indicates that patients with COVID-19 being investigated for VTE may be risk-stratified using conventional tools with consideration for disease severity^1–3^. On a system-level, our results indicate a clear need for continuous feedback on the prevalence of certain conditions, such as VTEs, so that the treating physician can make informed decisions when planning the workup of patients. Although a few additional radiological examinations may not seem like much to the individual practitioner, widespread increases in certain diagnostic modalities put significant strain on a healthcare system and potentially cause displacement effects for other patient groups, as well as substantial cost increases.

## Data Availability

Abstracted data is available from the corresponding author on reasonable request.
